# The more engaging, the more enjoyable? Age matters in predicting perceived enjoyment with different Facebook activities

**DOI:** 10.3389/fpsyg.2022.994337

**Published:** 2022-11-02

**Authors:** Lianshan Zhang, Eun Hwa Jung

**Affiliations:** ^1^School of Media and Communication, Shanghai Jiao Tong University, Shanghai, China; ^2^School of Media and Advertising, Kookmin University, Seoul, South Korea

**Keywords:** Facebook activities, younger adults, middle-aged adults, older adults, perceived enjoyment, uses and gratifications, socioemotional selectivity theory

## Abstract

Drawing upon uses and gratifications perspectives and socioemotional selectivity theory, this study examined the differences in the use of Facebook features among younger, middle-aged, and older adults. Furthermore, it explored the association between Facebook activities and users’ perceived enjoyment for different age groups. An online survey was conducted with 647 Facebook users in the United States. An exploratory factor analysis identified four types of Facebook activities: broadcasting, directed communication, content consumption, and information regulation. The results revealed that younger users’ broadcasting, content consumption, and information regulation activities substantially differed from those of older users. In addition, broadcasting and content consumption activities were more popular among younger users and more predictive of their enjoyment. Directed communication was more powerful in predicting middle-aged and older users’ enjoyment. However, younger and middle-aged users’ engagement with information regulation activities was negatively related to their enjoyment of Facebook. The study findings provide more nuanced knowledge regarding user experiences on social media platforms among specified age groups and practical insights into the improvement of social media by addressing their different needs.

## Introduction

Social media platforms have become increasingly popular and essential for daily communication across generations. According to Pew Research’s most recent social media fact sheet, 84% of United States adults between ages 19 and 29, 81% between ages 30 and 49, and 73% of adults between ages 50 and 64 use at least one social media platform (Pew Research Center, 2021). In addition, 50% of United States adults aged 65 years and above were Facebook users (Pew Research Center, 2021), indicating its popularity among older adults. The enhanced functionality of social media enables individuals to freely use a panoply of social media features that are beneficial for their well-being (Tamborini et al., 2010). Accordingly, examining social media use is essential to explore its great potential, thereby ensuring users’ positive feelings and beneficial experiences.

However, previous studies investigating the relationship between social media use and user experience indicated the following limitations: First, most of the extant research has considered social media use as a relatively monolithic activity, measuring it as a whole (i.e., time duration, use vs. nonuse). Furthermore, no fundamental differentiation exists regarding specific affordances (e.g., Yu et al., 2016). Thus, the theoretical understanding of the antecedents and outcomes of social media use remains limited as people utilize them for different purposes and pursue diverse activities (Smock et al., 2011). Second, prior studies examining social media use have primarily focused on adolescents and college students despite the massive increase in social media use by older adults in recent years (Faverio, 2022). In addition, social media use may particularly benefit older populations in dire need of social interaction, family connections, etc., especially those facing the challenges of daily functioning problems. Accordingly, examining the user experience of older adults, the most rapidly growing social media user group, is necessary and bears pragmatic importance. Third, given the major focus on adolescents and college students in previous research, studies highlighting the possible generational differences in the patterns of social media use and its varying effects have been scarce.

To address the aforementioned research gaps, this study examined Facebook use across age groups, with two objectives. First, unlike previous studies considering social media usage as a monolithic activity, this study analyzed the frequency of using diverse Facebook features that attract users from various age groups and the potential generational differences related to it. Second, understanding the type of social media features that benefits users and affords positive experiences specific to each age group is essential. According to Turel and Serenko (2012), perceived enjoyment is a salient positive experience of social media usage. The uses and gratifications (U&G) perspective contends that users are active and have considerable control in selecting and using media to fulfill their inner needs, such as enjoyment (Katz et al., 1973). In addition, socioemotional selectivity theory (SST) demonstrates that people at different life stages assign different values and priorities to distinct social motivations because of an age-associated shift in time perspective (Carstensen et al., 1999). Accordingly, the varying priority of social goals by different generations may be reflected by their use and interactions on Facebook, an essential part of people’s daily social networking. These different usage patterns involving unique sets of features may make a difference in users’ perceived enjoyment when using Facebook. Therefore, another objective of this study was to investigate the association between users’ Facebook activities and perceived enjoyment for distinct age groups. Following previous studies examining the use of information communication technologies (ICTs) from a lifespan perspective (e.g., Van Volkom et al., 2013; Chan, 2018), we examined the relation between Facebook use and perceived enjoyment for three age groups: younger users (18–34 years old), middle-aged users (35–59 years old), and older users (60 years old and above).

## Literature review

### Use of social media features

Social media represent major sources of information and facilitate daily communication through diverse features. For instance, Facebook enables users to directly communicate with their Facebook friends one-to-one through its messaging function and comments. It also allows them to broadcast messages to large audiences using timeline posts and status updates in a one-to-many style. Social media are multipurpose online services integrating a wide array of technical features from which users can select (Boyd and Ellison, 2007). Considering this perspective, scholars have challenged the way of investigating social media use by viewing it as a whole object without disaggregating the medium into specific variables (Burke and Kraut, 2016; Zhang et al., 2022). This approach treats interactive technology as a “black box.” To unlock this technological “black box” and better understand processes by which an aspect of technology impacts user psychology, Sundar and Bellur (2010) conceptualized technological attributes of interactive media as “affordances.” Affordances are denoted as action possibilities suggested by visual stimuli in our environment (Gibson, 1986). Accordingly, this study adopted an affordance-based approach to unpack activities allowed by specific features to better examine users’ affordance-enabled behaviors.

Previous studies have considered the utilization of specific social media features and have identified several types of activities. For instance, focusing on the features facilitating user communication, Smock et al. (2011) determined that users’ motivations predicted differential patterns of feature use (e.g., status updates, comments, and private messages) among college students. Burke et al. (2011) classified frequently used Facebook features into three social activities to further distinguish social behaviors on Facebook: directed communication with individuals’ friends, passive content consumption of social news, and broadcasting. Moreover, Frison and Eggermont (2016) applied an affordance-based approach and differentiated three types of Facebook use: passive use (e.g., reading news feed), active private use (e.g., chatting with someone on Facebook), and active public use (e.g., posting a message on the Facebook timeline).

Individuals with different goals and desires act differently on social media by using the features that enable diverse forms of interactions. However, prior studies have primarily targeted specific groups of users such as teens and college students (e.g., Frison and Eggermont, 2016; Roberts and David, 2020). Therefore, social media become geared toward younger generations, catering to their needs and user habits, and have overlooked the potential of older consumers, the most rapidly growing user group (Faverio, 2022). Hence, investigating the potentially different social media usage across age groups is imperative for providing a richer understanding of diversified user practices and experiences of social media.

### Social media use across age groups

Prior research has indicated that different age groups exhibit distinct motivations for and expectations of using social media platforms (Steijn, 2014). Theoretically, as a lifespan theory of motivation, SST states that individuals’ age-related time perspectives play a key role in prioritizing their goals and preferences for social communication (Carstensen et al., 1999). Specifically, younger adults have open-ended time horizons, leading them to prioritize future-oriented goals concerning the acquisition of new knowledge and information, career networking, and the establishment of new social ties that are preparatory and serve for their future. By contrast, older adults are increasingly aware of time limitations and prioritize present-oriented goals aimed at optimizing emotional gratification (Lang and Carstensen, 2002). These adaptive changes lead older adults to spend more time interacting with their close social bonds, from whom they derive the most emotional gains (Carstensen, 2006). More importantly, the U&G approach argues that people select media or particular media features based on motives that reflect their social and psychological needs (Katz et al., 1973; Smock et al., 2011). Integrating SST with U&G, the age-related differences in social motivations and preferences may be exhibited in the types of activities that users engage in online interactions.

Empirical studies have suggested that users’ age is critical in understanding their online social communication. Vroman et al. (2015) indicated that users’ activities based on ICTs (e.g., communicating *via* email with family and sending instant messages) were significantly associated with the perceived importance of these activities closely related to aging. Chang et al. (2015) extended the SST to online social networking. They determined that age was negatively associated with Facebook network size but positively associated with the proportion of actual Facebook friends. Furthermore, they indicated that age was negatively associated with self-posting and checking information. In addition, Ozimek and Bierhoff (2016) revealed that age was negatively associated with the frequency of the following Facebook activities: watching (inspecting information from others), impressing (self-presentation), and acting (performing an activity). McAndrew and Jeong (2012) demonstrated that younger adults are more actively engaged in posting profile pictures and seeking personal information about others. Thus, the findings of prior studies have suggested the need for examining potentially different user practices on social media across age groups.

However, the majority of prior studies have overlooked the diverse social media activities attracting both younger and older adults, as well as lacking clear evidence of specific usage patterns across age groups. Kim and Shen (2020) attempted to address these gaps by comparing Facebook use between younger (18–25 years) and older users (over 50 years) with regard to directed communication and broadcasting behavior. They identified that older adults were engaged in directed communication activities more frequently than younger adults; no difference was observed concerning broadcasting activities. However, Kim and Shen’s (2020) secondary data analyses were limited to one type of directed communication (tagging) and two broadcasting activities (status updates and photo uploads), and were susceptible to the potentially confounding variables that were not available for controlling for, such as socioeconomic status, general Facebook usage (e.g., time spent on Facebook), and power usage.

Overall, our discussion highlights a critical gap in the literature concerning the use of social media activities across age groups. Evidence suggests that younger adults differ from older populations in terms of social media use in general (e.g., McAndrew and Jeong, 2012; Ozimek and Bierhoff, 2016). However, research concerning the specific differences in social media activities across age groups remains inadequate. The lack of direct comparisons among age groups makes it difficult to assess the differences in social media use between younger and older individuals. Hence, we established the research question as follows:

RQ1: What is the difference in the use of Facebook features among younger, middle-aged, and older adults?

### Social media use and perceived enjoyment

Social media are pleasure-oriented platforms, and social media use has a self-fulfilling value (Reinecke et al., 2014). Social media offer more functions to users than they would initially adopt, and individuals usually choose a particular set of features that they think would best meet their personal needs (Leiner et al., 2018). This flexible nature of social media reflects the central premise of U&G that audiences are motivated by their inner needs in media selection and use. As such, according to SST, younger and older adults have different usage patterns on Facebook because of their respective prioritized social goals relating to age. For instance, Jung and Sundar (2016) indicated that social bonding was the primary motivation predicting older adults’ Facebook activities. They also found that posting on other people’s walls and Facebook chatting with others were the only two Facebook features that significantly constituted older adults’ time spent on Facebook. A systematic review of social media use among older adults revealed that they mainly utilize social media to maintain contact with close family and friends, whereas using it to form new connections appeared to be less important (Newman et al., 2019). By contrast, individuals in emerging and young adulthood have a greater need for identity formation and development of intimate relationships, thereby engaging more in social media activities concerning networking extensions and disclosure of personal information (Steijn, 2014; Van den Broeck et al., 2015). With such varying patterns of user practices and preferences on social media, users across age groups can potentially experience distinct levels of enjoyment as well.

As a positive experiential response, perceived enjoyment from engaging in social media is an intrinsic reflection of pleasurable experiences that arise from consuming media or performing a certain activity on a system (Turel and Serenko, 2012). In particular, U&G identifies enjoyment as one of need satisfaction (Katz et al., 1973), and individuals’ activities on social media can result in feelings of enjoyment (Reinecke et al., 2014). For example, the use of interactivity affordances on social media (e.g., messaging, sharing) offers users more opportunities to contact and share with their social network, creating a greater sense of connectedness, social presence, and pleasure (Cheikh-Ammar and Barki, 2016). Asynchronous functions (e.g., commenting) and customization features (e.g., profile customization) of social media provide users with greater control of nonverbal communication cues and content for communication that helps users show up selectively (Walther, 2011), giving them more pleasure and enjoyable experiences (Reinecke et al., 2014). Moreover, the consumption of user-generated content, such as shared photos, videos, and personal stories, ensures entertainment for individual users (Hart et al., 2008). In the literature on information systems, perceived enjoyment is considered a driving force behind positive system use, such as high engagement with the system and future usage intentions—especially those who used for communication purposes (Turel and Serenko, 2012). Given a broad set of social media features and the importance of enjoyment in improving positive user experiences with social media, examining how the levels of enjoyment are determined from different social media usages across user groups is essential, contributing to a more precise development of social media. Thus, we investigated the types of technological features on Facebook effective in inducing a feeling of enjoyment across age groups through the following research question:

RQ2: To what extent do the relations between the use of Facebook features and perceived enjoyment differ among younger, middle-aged, and older users?

## Materials and methods

### Data collection

To answer the research questions, this study conducted an online survey in September 2019 by employing convenience sampling *via* Qualtrics’s panels. Participants were randomly selected from Qualtrics’s panels. Upon participants’ consent to participate in the survey, they were asked whether they have used Facebook, and then, non-Facebook users were excluded. In total, 647 Facebook users in the United States were recruited. The sample comprised 156 (24.1%) males and 491 (75.9%) females, as well as 172 (26.6%) younger adults (18–34 years old), 270 (41.7%) middle-aged adults (35–59 years old), and 205 (31.7%) older adults (60 years old and above), with a mean age of 48.03 (SD = 15.89). Most respondents were Caucasian (80.4%, *N* = 520). Approximately 42.2% (*N* = 273) of respondents were married; more than half of the respondents (69.7%, *N* = 451) acquired some college degree or higher education, and less than half of the participants (40.5%, *N* = 262) reported an annual household income of $50,000 or more. It should be noted that in terms of the demographics of Facebook users in the United States, the most recent statistics show that 42% of Facebook users are aged 18–34 years old, 31.7% of users are aged 35–54 years old, and 21.8% of Facebook users are 55 years old and over (Statista, 2022a). Although this study recruited participants across all age groups, our data turned out to include more older users than the general Facebook users in the United States with more older Facebook users aged 55 and over and fewer younger users aged 18–34 years old. With regard to gender distribution, 46% of Facebook users in the United States are reported as female, and 54% of users are indicated as male (Statista, 2022b). Accordingly, our data consist of more females (75.9% vs. 46%) than general Facebook users in the United States. Additionally, compared with the most recent U.S. census data (United States Census Bureau, 2021), our study recruited a bit more Caucasians (80.4 vs. 75.8%) but fewer Blacks or African Americans (9.9 vs. 13.6%).

### Measures

An online questionnaire measured Facebook activities, perceived enjoyment, power usage, general Facebook usage, demographics, and other control variables. Multiple-item measurements were adopted from previously established literature to assure the content and construct validity. The measurement scales employed in our study also achieved acceptable reliability greater than 0.70 (Cronbach, 1951).

#### Facebook activities

To examine the extent to which participants use specific Facebook features, we included 25 frequently used Facebook activities identified by previous research (Burke et al., 2011; Jung and Sundar, 2016; Leiner et al., 2018). Respondents were asked to rate their own frequency of use for each activity on a 7-point scale (1 = *never*, 7 = *very frequently*; see Table 1 for the full list of activities).

**Table 1:** Factor analysis of Facebook activities.

	Factors					
Facebook activities	*M*	SD	1	2	3	4
**Factor 1: Broadcasting**	2.525	1.253				
1. Creating public pages	2.002	1.585	**0.779**	0.078	0.085	0.154
2. Posting Live videos	2.034	1.613	**0.777**	0.154	0.099	0.124
3. Creating a Facebook group	1.985	1.510	**0.773**	0.087	0.132	0.209
4. Creating an event on Facebook	2.256	1.709	**0.765**	0.157	0.112	0.172
5. Posting 360 (degree) photos on your own Facebook timeline	1.912	1.539	**0.736**	0.141	0.053	0.185
6. Changing items on your Facebook profile (e.g., work and education, basic information, details about you, etc.)	2.698	1.578	**0.667**	0.244	0.207	0.111
7. Using “check in” to update your location	2.536	1.772	**0.656**	0.297	0.221	0.076
8. Posting videos on your own Facebook timeline	2.991	1.870	**0.526**	0.391	0.243	0.199
9. Changing your profile photo	3.297	1.717	**0.511**	0.378	0.322	0.074
10. Updating your status (“what’s on your mind?”)	3.543	1.854	**0.506**	0.396	0.254	−0.016
**Factor 2: Directed communication**	4.267	1.404				
11. Commenting on others’ status (“what’s on your mind?”)	4.297	1.777	0.196	**0.800**	0.154	0.045
12. Commenting on others’ photos	4.414	1.710	0.232	**0.796**	0.168	0.065
13. Replying to others’ comments on your profile photo, new photos, fan status, “what’s on your mind” status, group status, notes, and links	4.130	1.854	0.179	**0.732**	0.271	0.053
14. Clicking reactions (i.e., Like, Love, Haha, Wow, Sad, Angry) for a tie’s posts that you viewed	4.711	1.975	−0.018	**0.687**	0.276	0.089
15. Sending private messages through Facebook message function	4.386	1.915	0.161	**0.620**	0.204	0.123
16. Posting on other people’s Facebook timelines	3.662	1.714	0.328	**0.615**	0.144	0.087
**Factor 3: Content consumption**	4.363	1.433				
17. Checking out other people’s photos without leaving comments	4.564	1.689	0.141	0.152	**0.773**	0.052
18. Checking out people’s timelines without leaving a message	4.203	1.810	0.131	0.175	**0.765**	0.129
19. Checking out news feed	4.505	2.029	0.167	0.322	**0.671**	0.059
20. Viewing photos in news feed	4.408	1.916	0.148	0.375	**0.656**	0.182
21. Watching videos	4.133	1.839	0.248	0.393	**0.533**	0.181
**Factor 4: Information regulation**	2.602	1.477				
22. Hiding all posts (stop seeing posts from this page)	2.665	1.717	0.272	0.108	0.126	**0.837**
23. Hiding someone’s post (“see fewer posts like this”)	2.929	1.784	0.223	0.176	0.196	**0.815**
24. Snoozing someone for 30 days (temporarily stop seeing posts)	2.213	1.741	0.390	0.054	0.104	**0.598**
Eigenvalue			5.155	4.108	2.982	2.061
Variance explained (%)			22.413	17.863	12.965	8.959
Cronbach’s alpha			0.906	0.862	0.829	0.800

Factors determined by item loading of.5 or higher and no cross-loadings higher than 0.5.

#### Perceived enjoyment

Adapted from previous studies (Lee et al., 2012), three items were used to estimate the degree of enjoyment and pleasure that users perceived when using Facebook: (1) I find Facebook to be enjoyable, (2) The actual process of using Facebook is pleasant, and (3) I have fun using Facebook. The responses were anchored on a 7-point Likert scale (1 = *strongly disagree*, 7 = *strongly agree*), and the average of these three items was created for the composite scale of perceived enjoyment (*M* = 5.33, SD = 1.27, *α* = 0.93).

#### Power usage

Previous literature indicates that power users typically make productive use of different technological gadgets to explore and experience the technological options to the fullest extent (Sundar and Marathe, 2010; Zhong, 2013). They are generally self-motivated in exploring and adopting newer technologies and interface features and are more likely to employ features to control their interaction with technology (Sundar and Marathe, 2010). As a concept captures a user’s motivation (e.g., love to use technological gadgets), expertise (e.g., making good use of most features), efficacy (e.g., like to figure out how to use new technology), and demonstration of evolved technology use (e.g., tend to explore all available features), power usage was found to be significantly related to Facebook usage in prior studies (e.g., Jung and Sundar, 2018). In light of prior literature, this study included power usage as a control variable in an attempt to eliminate alternative explanations. To estimate the degree to which participants perceive their own ability to manage technologies in general, 12 items were adapted from the study by Sundar and Marathe (2010) on a 7-point Likert-type scale (e.g., “I make good use of most of the features available to me in any technological device”; *M* = 4.48, SD = 1.06, *α* = 0.77).

#### Overall Facebook use

Respondents were asked to indicate how many minutes were spent using Facebook per day to estimate their general use of Facebook. In addition, Facebook network size was measured by asking participants to report approximately the number of friends they have on Facebook. We found a heavily skewed distribution of the time spent on Facebook (*M* = 67.44, *Mdn* = 30.00, SD = 102.28, Skewness = 4.17); therefore, the scores were transformed using the natural logarithm (*M* = 3.48, *Mdn* = 3.40, SD = 1.28, Skewness: −0.38). Similarly, as the number of Facebook friends was highly skewed (*M* = 402.78, *Mdn* = 185, SD = 790.78, Skewness = 5.73), the scores were transformed for further analyses (*M* = 5.06, *Mdn* = 5.25, SD = 1.45, Skewness: −0.37).

#### Demographics

Social and demographic characteristics, including gender, ethnicity, education, income, and marital status were measured as controls.

## Results

### Exploratory factor analysis (EFA): Different Facebook activities

To examine the possible user patterns for a distinct Facebook feature, we conducted EFA on all 25 items with principal component extraction and varimax rotation to identify the underlying domains of Facebook activities [Kaiser–Meyer–Olkin (KMO) = 0.95]; Bartlett’s test of sphericity, [*χ*^2^(276) = 8283.53, *p* < 0.001]. The EFA yielded four factors, together accounting for 62.20% of the variance. The item of tagging on photos was dropped as it had significant cross-loadings on two factors, failing to meet the factor purity criteria (Tabachnick and Fidell, 2013).

The first factor, “broadcasting,” accounted for 22.41% of the variance (eigenvalue = 5.16), with 10 items (e.g., creating public pages and updating your status). The second factor, “directed communication,” accounted for 17.86% of the variance (eigenvalue = 4.11), with six items (e.g., commenting on others’ status and sending private messages). The third factor, “content consumption,” accounted for 12.97% of the variance (eigenvalue = 2.98), with five items (e.g., checking out people’s timelines without leaving a message and watching videos). The fourth factor, “information regulation,” accounted for 8.96% of the variance (eigenvalue = 2.06), with three items (e.g., hiding someone’s post, snoozing someone for 30 days). For further analyses, scales were created by averaging the ratings of all items that represented the respective factors. All scales indicated acceptable levels of reliability (broadcasting, *α* = 0.91; directed communication, *α* = 0.86; content consumption, *α* = 0.83; and information regulation, *α* = 0.80). Table 1 presents a full list of all items and factor loadings for the four types of Facebook activities.

### Differences in Facebook activities for three age groups

To examine RQ1, a series of analysis of covariance (ANCOVA) was conducted, which revealed that the engagement with Facebook activities was substantially different between younger and older users as well as between middle-aged and older users; no significant difference was observed between young and middle-aged users. Specifically, for broadcasting activities, when controlling for covariates, there was a significant relationship between age groups and the frequency of engaging in broadcasting activities on Facebook [*F* (2, 636) = 13.372, *p* < 0.001, *partial η*^2^ = 0.047]. Post-hoc comparisons using the Bonferroni test at alpha = 0.05 revealed that younger adults were more frequently engaged in broadcasting (*M* = 2.811, SD = 1.368) than older adults (*M* = 1.868, SD = 0.837). In addition, middle-aged users (*M* = 2.571, SD = 1.303) participated in more broadcasting activities than older users (see Table 2).

**Table 2:** ANCOVA results for different Facebook activities by age groups.

	Broadcasting		Directed communication		Content consumption		Information regulation	
	Mean difference (SE)	Bonferroni adjusted 95% CI	Mean difference (SE)	Bonferroni adjusted 95% CI	Mean difference (SE)	Bonferroni adjusted 95% CI	Mean difference (SE)	Bonferroni adjusted 95% CI
*Comparisons*								
Younger vs. Middle-aged	0.099 (0.118)	[−0.184, 0.383]	−0.283 (0.135)	[−0.608, 0.042]	0.262 (0.133)	[−0.057, 0.581]	0.164 (0.146)	[−0.186, 0.514]
Younger vs. Older	0.617^***^ (0.137)	[0.289, 945]	−0.093 (0.157)	[−0.469, 284]	0.620^***^ (0.154)	[0.250, 0.989]	0.713^***^ (0.169)	[0.308, 1.119]
Middle-aged vs. Older	0.518^***^ (0.112)	[0.249, 0.786]	0.191 (0.128)	[−0.117, 0.499]	0.358^*^ (0.126)	[0.055, 0.660]	0.549^***^ (0.138)	[0.217, 0.881]
*Covariates*	*F*		*F*		*F*		*F*	
Gender	7.439^**^	–	9.989^**^	–	4.445^*^	–	3.056	–
Ethnicity	2.354	–	0.150	–	0.013	–	0.560	–
Education	0.574	–	0.317	–	1.870	–	0.119	–
Income	1.045	–	0.045	–	0.079	–	2.291	–
Marital status	1.675	–	0.006	–	0.075	–	0.275	–
Facebook network size	1.403	–	1.748	–	5.148^*^	–	0.020	–
Time spent on Facebook	2.950	–	37.479^***^	–	37.830^***^	–	4.649^*^	–
Power usage	36.591^***^	–	34.076^***^	–	38.113^***^	–	9.540^**^	–

* *p* < 0.05;

** *p* < 0.01;

*** *p* < 0.001.

*R*^2^ for broadcasting = 0.177 (adjusted *R*^2^ = 0.164); *R*^2^ for directed communication = 0.138 (adjusted *R*^2^ = 0.124); *R*^2^ for content consumption = 0.202 (adjusted *R*^2^ = 0.189); *R*^2^ for information regulation = 0.095 (adjusted *R*^2^ = 0.080); Comparisons based upon analysis of covariance (ANCOVA) adjusted means controlling for covariates. CI = confidence interval.

However, no significant difference of engaging in directed communication activities were observed across three age cohorts [*F* (2, 636) = 2.605, *p* = 0.075, *partial η*^2^ = 0.008].

In terms of content consumption on Facebook, users across age groups displayed significantly different usage patterns [*F* (2, 636) = 8.345, *p* < 0.001, *partial η*^2^ = 0.026]. Specifically, younger adults were more frequently engaged in content consumption activities (*M* = 4.893, SD = 1.332) than older adults (*M* = 3.823, SD = 1.371). Moreover, middle-aged users (*M* = 4.434, SD = 1.404) participated in more content consumption activities than older users.

Regarding information regulation, when controlling for covariates, the results revealed a significant relationship between age groups and the use of information regulation features on Facebook [*F* (2, 636) = 10.764, *p* < 0.001, *partial η*^2^ = 0.033]. Post hoc comparisons indicated that younger adults were more likely to use information regulation features (*M* = 2.994, SD = 1.540) than older adults (*M* = 2.088, SD = 1.191); middle-aged users (*M* = 2.743, SD = 1.528) regulated more incoming content than older users.

### Different Facebook activities and their relationship with perceived enjoyment for three age groups

We ran three separate hierarchical regressions to examine specific types of Facebook activities that contribute to users’ enjoyment with Facebook for the three age groups. As summarized in Table 3, content consumption was the strongest predictor of younger adults’ enjoyment on Facebook (*β* = 0.323, *p* < 0.001), followed by broadcasting activities (*β* = 0.283, *p* < 0.01) and directed communication (*β* = 0.186, *p* < 0.05). However, the more frequently users employed the features for information regulation, the lower their levels of perceived enjoyment (*β* = −0.199, *p* < 0.05). For middle-aged adults, Facebook features concerning directed communication were the most powerful in predicting their feeling of pleasure (*β* = 0.428, *p* < 0.001), followed by broadcasting activities (*β* = 0.174, *p* < 0.01). Information regulation activities used for controlling the number of incoming posts were negatively related to middle-aged users’ enjoyment on Facebook (*β* = −0.203, *p* < 0.01). Content consumption on Facebook did not exhibit a significant relation to middle-aged users’ enjoyment (*β* = 0.007, *p* = 0.913). For older adults aged 60 years and above, the more they engaged in directed communication on Facebook, the higher their perceived enjoyment on Facebook (*β* = 0.391, *p* < 0.001). Conversely, compared with middle-aged adults, content consumption activities were significantly associated with older users’ enjoyment on Facebook (*β* = 0.229, *p* < 0.01). However, broadcasting (*β* = −0.089, *p* = 0.282) and information regulation activities (*β* = −0.064, *p* = 0.330) were not significantly associated with older adults’ pleasure on Facebook.

**Table 3:** Hierarchical regression results for different activities on perceived enjoyment on Facebook across different age cohorts.

	Age groups		
	Younger adults (*n* = 172)	Middle-aged adults (*n* = 270)	Older adults (*n* = 205)
*Block 1: Demographics*			
Gender (0 = male, 1 = female)	−0.004	0.072	−0.025
Ethnicity (0 = non-Caucasian, 1 = Caucasian)	0.013	0.015	0.011
Education	−0.153^*^	−0.044	−0.056
Income	0.040	−0.026	−0.002
Marital status (0 = non-married, 1 = married)	0.118	−0.067	−0.065
Block 1 Δ*R*^2^	0.025	0.017	0.012
*Block 2: General use*			
Facebook network size	−0.029	0.090	0.001
Time spent on Facebook	0.135^*^	0.079	0.094
Power usage	0.121	0.146^**^	0.169^*^
Block 2 Δ*R*^2^	0.112^***^	0.149^***^	0.145^***^
*Block 3: Activities*			
Broadcasting	0.283^**^	0.174^*^	−0.089
Directed communication	0.186^*^	0.428^***^	0.391^***^
Content consumption	0.323^***^	0.007	0.229^**^
Information regulation	−0.199^*^	−0.203^**^	−0.064
Block 3 Δ*R*^2^	0.323^***^	0.196^***^	0.182^***^
*R* ^2^	0.461^***^	0.362^***^	0.339^***^
Total adjusted *R*^2^	0.420^***^	0.332^***^	0.297^***^

* *p* < 0.05;

** *p* < 0.01;

*** *p* < 0.001.

Figures are standardized beta-coefficient. Beta weights are from final regression equation with all blocks of variables in the model; *N* = 647.

**Figure 1:**
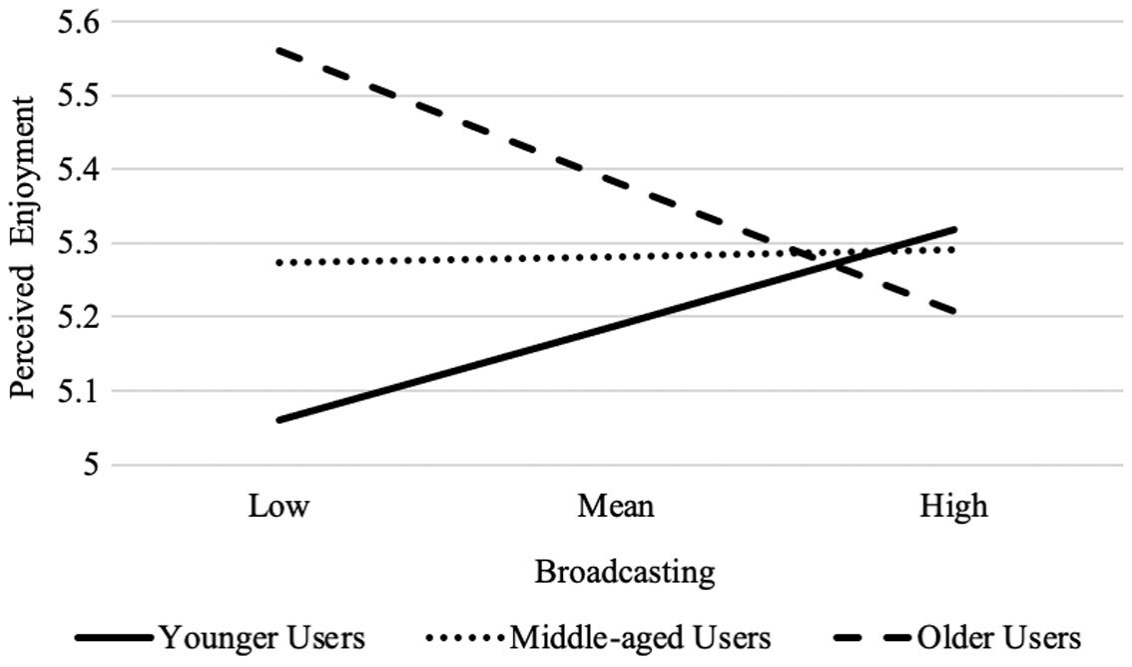
Moderating effect of user’s age on the relation between broadcasting activities and perceived enjoyment on Facebook; the higher value is one standard deviation above the mean, and the lower value is one standard deviation below the mean.

The macro PROCESS Model 1 with 5,000 bootstrap samples was employed to further probe how the relation between different Facebook activities and perceived enjoyment varied by age (Hayes, 2017). The results revealed a significant interaction effect of user’s age and broadcasting activities on perceived enjoyment: *b* = −0.005, se = 0.002, *p* = 0.005, 95% CI [−0.009, −0.002]. As illustrated in Figure 1, the positive relation between broadcasting behavior and Facebook enjoyment was more salient among younger Facebook users, *b* = 0.079, se = 0.037, *p* = 0.023, 95% CI [0.006, 0.152], but not among middle-aged users, *b* = −0.007, se = 0.035, *p* = 0.685, 95% CI [−0.076, 0.061], and older users, *b* = −0.094, se = 0.053, *p* = 0.071, 95% CI[−0.199, 0.010].

**Figure 2:**
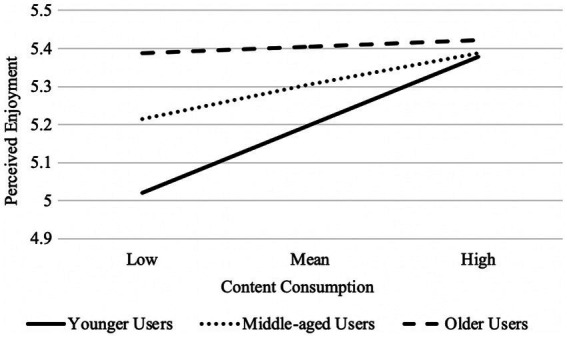
Moderating effect of user’s age on the relation between content consumption and perceived enjoyment of Facebook; the higher value is one standard deviation above the mean, and the lower value is one standard deviation below the mean.

In addition, a significant interaction effect was observed of age and content consumption on perceived enjoyment, *b* = −0.004, se = 0.002, *p* = 0.012, 95% CI [−0.007, −0.001]. As illustrated in Figure 2, the positive association between content consumption and perceived enjoyment was more apparent among younger users, *b* = 0.140, se = 0.038, *p* = 0.000, 95% CI [0.065, 0.214], but not among middle-aged users, *b* = 0.063, se = 0.038, *p* = 0.087, 95% CI [−0.012, 0.138], and older users, *b* = 0.027, se = 0.038, *p* = 0.483, 95% CI [−0.048, 0.101].

However, no significant interaction effect was observed of users’ age and the frequency of engaging in directed communication on perceived enjoyment, with *b* = −0.002, se = 0.002, *p* = 0.160, 95% CI [−0.005, 0.001]. Furthermore, no interaction effect was observed of users’ age and the engagement with information regulation activities on perceived enjoyment, *b* = −0.002, se = 0.001, *p* = 0.223, 95% CI [−0.005, 0.001].

## Discussion

Contextualized in an ongoing online social networking landscape across generations, this study identified different Facebook activities and examined their different usage across three age groups. Our findings revealed substantially diversified practices contingent upon age. It appears that different age groups act in their unique ways for deriving feelings of enjoyment from Facebook.

### Different Facebook activities

This study identified four Facebook activities that attract both younger and older users: broadcasting, directed communication, content consumption, and information regulation. Although our study included a broader set of features, this study’s categorization of different Facebook activities is generally consistent with the characterization of Facebook features in previous research (e.g., Burke et al., 2011; Verduyn et al., 2015). In addition to the three well-categorized Facebook activities (broadcasting, directed communication, and content consumption), our results uncover an additional type of Facebook activity that allows users to regulate their incoming content for personal consumption, namely information regulation. Compared with the other three types of Facebook activities, activities related to information regulation are relatively understudied in previous research; however, nowadays, people increasingly experience information overload on social media (Matthes et al., 2020), and these activities have the potential to reduce overwhelming feelings and aid individuals’ regulation of “inward-facing territories” (Wisniewski et al., 2012, p. 610). The information regulation activity that captures users’ curatorial practices of filtering and selecting preferred content from one’s news feed echoes the concept of news curation or consumptive news feed curation in the literature of personalization in the context of social media use (Lu, 2020; Merten, 2021). The pertinent discussion derived from our results adds to the current scholarship on customization and selective exposure on Facebook and other algorithmic platforms by considering users’ age and associated social needs from the theoretical perspective of SST.

### Facebook activities and perceived enjoyment across age groups

Of the four underlying domains of Facebook activities, this study revealed that users in different age groups exhibited various user practices concerning broadcasting, content consumption, and information regulation activities. Along with this, users in different age groups experienced feelings of enjoyment on Facebook disparately.

First, younger users were engaged more in broadcasting acts and tended to self-disclose a larger amount of personal information on Facebook than older adults; these results are consistent with that of prior studies (Steijn, 2014; Van den Broeck et al., 2015). This usage pattern may be closely related to the central psychosocial task of young adulthood, such as identity formation and network extensions (Erikson, 1959; Steijn, 2014). In light of the literature on developmental trajectories, young adults are in great need of self-presentation and seek to expand social horizons outside their core family members to accomplish developmental tasks such as career entry and marriage (Wrzus et al., 2013). Given such psychosocial needs, broadcasting activities—such as sharing personal stories and revealing them selectively with a large group of social media users—is an effective way of presenting self-focus information to a broader audience and a catalyzer of social interactions with a wider audience, fostering the younger population’s self-presentation, identity construction, and new relationship development (McAndrew and Jeong, 2012; Steijn and Schouten, 2013). This also explains why younger users largely obtain enjoyment from broadcasting activities, in line with prior studies (Wise et al., 2010).

In addition, our results revealed that middle-aged users participated in more broadcasting activities on Facebook compared to older users. They also garnered need satisfaction from broadcasting. Studies indicated that middle-aged adults are busy with both family and social lives because they manage a mixed social world, needing to strengthen bonds with existing ties (e.g., old friends and family) while also committing to nurturing new contacts for career development or professional advancement (Van den Broeck et al., 2015). Middle-aged people seek to strengthen their social identity and pursue self-actualization through family and/or work (Waterman, 1982; Hardy and Castonguay, 2018). Combining these social developmental goals with the great potential of broadcasting activities in cultivating maintained and bridging social capital (Ellison et al., 2007) that fosters the achievement of these goals (Lin, 1999), our results imply that broadcasting activities are conducive to middle-aged users’ social needs gratification in social media contexts. The direct comparison of Facebook use among the three age groups suggests a general trend by which the significant decline of broadcasting activities begins from older adulthood rather than from middle adulthood—although it may start to shift during middle adulthood.

Second, younger and middle-aged users were engaged more in content consumption activities than older users. Although content consumption does not involve direct exchanges with others, browsing and viewing Facebook content functions as a means of social information acquisition and monitoring, which allows users to keep abreast of the happenings and novel events in their larger social circles (Burke et al., 2011; Zhang et al., 2022). This social monitoring is beneficial for broadening horizons and is valuable in gratifying younger users’ social bridging needs (Ellison et al., 2007); therefore, in addition to broadcasting activities, they mainly pursued enjoyment from content consumption.

Noticeably, the results indicated that although older users were less frequently engaged in content consumption on Facebook than their younger compatriots, they gained enjoyment from such activity. On the one hand, the finding resonates with the demonstration that the media with which an audience grows up influence the way they perceive and consume media as they age (Valentine, 2013). Older generations are familiar with traditional, one-to-many media formats consumption such as television viewing and newspaper reading. This traditional media consumption style is similar to content consumption on social media; therefore, older adults tend to experience more pleasure from their familiar consumption habits. On the other hand, the finding that the elderly garnered enjoyable experiences from content consumption aligns with the results of previous studies. These studies have indicated that older adults more easily gather positive platform experiences from easy and convenient features that require a low level of cognitive effort (Mitzner et al., 2010) because of a limited technological proficiency (Czaja et al., 2006).

Third, our results revealed that users in different age groups did not differ with regard to directed interpersonal communication; this explains, at least in part, the popularity of using directed communication activities on Facebook for daily interactions across generations. This explanation was further supported by our finding that users in all of ages reap enjoyment from directed communication. Nevertheless, by taking a closer look at the disparate beta coefficients, it seems that directed communication is the primary source of enjoyment for middle-aged and older users. This is not true for younger users, who largely gained pleasure from broadcasting and consumption. This result is reasonable in light of SST; people in middle and late adulthood are keen to maximize the emotional payoffs so as to derive meaningful and positive experiences from life (Carstensen et al., 1999), and directed communication through targeted message exchanges with multimedia content can strengthen intimate relationships (Burke and Kraut, 2016), the source of emotional gratifications. As such, pursuing and obtaining fulfillment of emotional needs through directed communication on Facebook can greatly contribute to middle-aged and older users’ enjoyable experiences on Facebook.

In terms of information regulation, we found that younger and middle-aged users frequently managed their incoming information on Facebook than older users, in accordance with the findings of previous research (Van den Broeck et al., 2015; Ozimek and Bierhoff, 2016). The lower usage of information regulation activities among older users may be due to the lower awareness of the settings or the lack of digital literacy and efficacy in using relatively newer features on social media (Van Volkom et al., 2014; Lu, 2020). In fact, studies have documented that despite the behavioral intentions of hiding people and information on Facebook, users’ actual behaviors were deterred by the absence of necessary knowledge and skills (e.g., Koroleva et al., 2010). Additionally, older users were found to have higher privacy concerns but lower privacy settings on Facebook than younger users because of the lack of knowledge about how to adapt relevant settings (e.g., Van den Broeck et al., 2015). Furthermore, the frequent usage among younger and middle-aged users to avoid exposure to certain information (e.g., updates from certain friends, acquaintances, and pages) may signal the emerging phenomenon of information overload among younger and middle-aged users.

More importantly, the results revealed a negative association between information regulation activities and enjoyment among younger and middle-aged users. Consistent with prior studies (e.g., Peña and Brody, 2014), information regulation activities, such as hiding a Facebook contact or his or her posts, are associated with users’ negative emotions and poor user experiences that are likely triggered by negative interactions (e.g., impolite and rude status updates, socially inappropriate postings). On the one hand, younger and middle-aged users have been found to have a large number of peripheral Facebook friends—acquaintances and strangers (Koroleva et al., 2010; Chang et al., 2015). These friends may have different worldviews from users and their posts may be irrelevant, tactless, insensitive, or threaten the receivers’ self-concept and beliefs; thus, they are more susceptible to arousing users’ avoidance actions and related negative feelings (Weinert et al., 2012; Peña and Brody, 2014). By contrast, as older users have a higher proportion of “actual” friends on social media (Chang et al., 2015; Yu et al., 2018), composing primarily of emotionally close social partners (e.g., children and grandchildren), they are less likely to encounter unwanted postings and social chaos from peripheral contacts, the main impetus of information regulation activities and related unpleasant experiences on Facebook (Weinert et al., 2012; Wisniewski et al., 2012). On the other hand, younger and middle-aged users have evinced larger networks with greater encounters of excessive streams of incoming information (e.g., Choi and Lim, 2016). Such an overwhelming information environment can elicit information overload in younger users, leading up to their feelings of social media fatigue and displeasure with Facebook use (Gao et al., 2018; Fu and Li, 2022). In this sense, the negative relationship between younger and middle-aged users’ frequent engagement with information regulation and perceived enjoyment may suggest that younger and middle-aged users tend to adopt information regulation activities as coping strategies to change the settings of Facebook’s algorithms to avoid psychological discomfort from information overload, as per prior research (e.g., Wisniewski et al., 2012).

Lastly, it is worth noting that although we found that middle-aged users can gain pleasure from broadcasting behavior and older users indeed felt enjoyable from content consumption, the interaction effects suggest that these Facebook practices are still more rewarding to the younger population than their older counterparts, pertaining to positive user-platform experiences. This gap highlights the need to improve the design of social media platforms, particularly considering middle-aged and older users’ needs, expectations, and concerns.

### Theoretical and practical implications

This study uses a modern U&G approach in social media contexts across generations to provide new insights for future research. First, in responding to growing concerns about measuring social media use as a monolithic entity (Frison and Eggermont, 2016; Leiner et al., 2018), this study examined Facebook use through an affordance-based approach and reinforced the necessity of differentiating Facebook activities to understand user practices and experiences. In addition, individuals usually perform a series of related actions that serve similar functions in deriving certain gratifications from social media (Leiner et al., 2018); hence, it is important to unearth underlying patterns or chains of every single act on the platforms. The four types of Facebook activities discovered from this study help transform specific features’ uses into meaningful concepts for grasping the underlying motives and social processes of social media activities. This is an important step to clearly identify user practices and expected gratifications that transcend specific social media platforms. Considering that certain features are platform-specific, the finding also contributes to the understanding of general user patterns that may exhibit on a broad range of applications from other providers.

Second, while previous research has applied SST when explaining the differences of social media network composition between younger and older users (e.g., Chang et al., 2015; Yu et al., 2018), this study synthesizes U&G with SST to elucidate the uses of Facebook features in terms of pursuing different social and emotional gratifications across different age groups. In particular, by conceptualizing perceived enjoyment as an important need for satisfaction (Tamborini et al., 2010; Reinecke et al., 2014), this study demonstrates that dynamic intrinsic needs relating to developmental changes, which people undergo with age, can also reflect in their online social media behaviors; this adds to the understanding of people’s online practices concerning generational differences from a U&G perspective. Our findings of the differential Facebook activities across the three age groups suggest that the U&G is not a conclusive theory, opening up future research trajectories in integrating U&G with other motivational theories or developmental perspectives in the investigation of newer media uses and gratifications across generations.

The findings of this study provide several practical implications. For example, we found that monitoring the digital footprints of others and broadcasting personal information on Facebook are common among young users, who experience enjoyment largely through these two types of activities. Accordingly, in addition to encouraging younger users to produce and distribute diverse forms of user-generated content, social media practitioners could consider providing additional opportunities for users to express themselves reflecting their values and perspectives on the social media interface. Specifically, when users share some articles or video clips on their Facebook timelines, they can be invited to evaluate the quality or relevance of the shared content to their close social media friends with rankings of stars or scores to better express opinions and attitudes. Furthermore, given the finding of a negative relation between regulating incoming content for personal consumption and perceived enjoyment, the social media system should employ adaptive algorithms to diagnose the kind of content that seems to provoke hiding and snoozing activities; moreover, it should customize the content displayed on Facebook’s News Feed by updating it to offer younger users’ a favorable communication context for consumption.

In addition, social media designers need to develop features that prompt directed interpersonal communication by considering the enjoyment experiences of the three age groups. An social media platform, for instance, could send notifications of relevant posts from members of a “privileged” social network and suggest appropriate actions and courteous responses to the users to spur their direct communication. Moreover, alerts of family members’ postings and reminders of key pictures or posts could be prioritized to foster potential intergenerational communication.

Finally, content consumption of a social tie’s information on Facebook functions as a fulfilling leisure activity for older users. Given that older adults prefer easy and convenient social media features (Mitzner et al., 2010), the finding signifies the importance of designing easy-to-learn features and intuitive settings to mitigate the complexity of the interface. In particular, additional training sessions should be held to advance the digital skills of older users by considering the low-level usage of newer features (e.g., hiding and snoozing). For instance, when introducing some new features, providing older populations with more tutorials, such as short videos that feature its purpose and specific hands-on procedures, is recommended to stimulate their interests and help them grow accustomed to the changes.

### Limitations and suggestions for future research

Several limitations are worth examining in future research. Although prior U&G studies have indicated that users are self-aware enough to recall and report their media use (Katz et al., 1973; LaRose et al., 2001), the first limitation that must be mentioned is the use of self-reporting measures. Measuring power usage may be susceptible to biased self-evaluation of one’s capabilities in managing technologies. We also recommend that future research use activity log data to more precisely measure social media activities and validate our findings. Another methodological limitation concerns the convenience sampling employed in this study. Although this study controlled for a set of demographic variables (e.g., gender, ethnicity, education, income, and marital status) in our statistical analyses, the interpretation of our results should note the bias of the non-probability sampling, considering certain over-represented demographic groups in our study (e.g., females and Whites). Future research is recommended to employ probability sampling with nationally representative samples to ensure broader generalizability of study findings. Third, although our study attempted to understand user practices in light of the motivational mechanisms across lifespans guided by SST, we did not directly assess social and emotional goals across different age groups. Future studies could include such measurements to directly examine the relationship between developmental goals and social activities on social media across generations. Finally, this study identified certain types of features that afford positive platform experiences to users; future research should consider the circumstances under which the enjoyable experience is intensified or discouraged by specific types of features. This will provide a richer explanation about why and how certain social media features usage provides users’ need satisfaction.

## Conclusion

Online social networking permeates almost every aspect of daily life. Understanding diverse social media activities and their relationships with users’ enjoyment experiences across generations is crucial to maximizing the benefits of social media for the quality of life of all ages. This study sheds light on the need for comprehensive research on social media user experience by considering different social media features and their usage by age groups, providing nuanced knowledge of user experiences across different age groups, as well as social media design recommendations based on users’ needs across their lifespan.

## Data availability statement

The raw data supporting the conclusions of this article will be made available by the authors, without undue reservation.

## Ethics statement

The studies involving human participants were reviewed and approved by the study protocol was approved by the Department of Ethics Review Committee at the National University of Singapore. The patients/participants provided their written informed consent to participate in this study.

## Author contributions

LZ and EJ contributed to conception and design of the study. LZ wrote the first draft of the manuscript. EJ collected the research data. All authors contributed to the article and approved the submitted version.

## Funding

This research is partly supported by the Science and Technology Commission of Shanghai Municipality [grant number 22692191400], and sponsored by Shanghai Pujiang Program [grant number 22PJC062].

## Conflict of interest

The authors declare that the research was conducted in the absence of any commercial or financial relationships that could be construed as a potential conflict of interest.

## Publisher’s note

All claims expressed in this article are solely those of the authors and do not necessarily represent those of their affiliated organizations, or those of the publisher, the editors and the reviewers. Any product that may be evaluated in this article, or claim that may be made by its manufacturer, is not guaranteed or endorsed by the publisher.
